# The collateral caval shunt as an alternative to classical shunt procedures in patients with recurrent duodenal varices and extrahepatic portal vein thrombosis

**DOI:** 10.1186/2047-783X-19-36

**Published:** 2014-06-25

**Authors:** Hans Michael Hau, Peter Fellmer, Markus B Schoenberg, Moritz Schmelzle, Mehmet Haluk Morgul, Felix Krenzien, Georg Wiltberger, Albrecht Hoffmeister, Sven Jonas

**Affiliations:** 1 Department of Visceral, Transplantation, Vascular and Thoracic Surgery, University Hospital of Leipzig, Liebigstrasse 20, 04103 Leipzig, Germany; 2 Department of Internal Medicine, Gastroenterology, University Hospital of Leipzig, Liebigstrasse 20, 04103 Leipzig, Germany; 3 Department of Visceral, Transplantation, Vascular and Thoracic Surgery, University Hospital of München (LMU), Marchioninistrasse 15, 81377 Munich, Germany

**Keywords:** duodenal varice, portal vein thrombosis, portal hypertension, shunt surgery, collateral caval shunt

## Abstract

Upper gastrointestinal bleeding episodes from variceal structures are severe complications in patients with portal hypertension. Endoscopic sclerotherapy and variceal ligation are the treatment options preferred for upper variceal bleeding owing to extrahepatic portal hypertension due to portal vein thrombosis (PVT). Recurrent duodenal variceal bleeding in non-cirrhotic patients with diffuse porto-splenic vein thrombosis and subsequent portal cavernous transformation represent a clinical challenge if classic shunt surgery is not possible or suitable.

In this study, we represent a case of recurrent bleeding of duodenal varices in a non-cirrhotic patient with cavernous transformation of the portal vein that was successfully treated with a collateral caval shunt operation.

## Background

Related to continuing improvements in endoscopic, interventional radiological and pharmacological treatment options, therapy strategies for cirrhotic and non-cirrhotic patients suffering from portal hypertension-related complications have undergone rapid change within the last years [1,2].

Management of disease complications such as varices of the gastrointestinal system plays an important role in therapy progress [3,4].

Compared to gastric and esophageal varices, duodenal varices are rarely found in patients with (extra-) hepatic portal hypertension due to portal vein thrombosis (PVT). Therefore, in this patient base, bleeding complications often entail much more serious and fatal consequences [5,6].

Endoscopic sclerotherapy and endoscopic variceal ligation replaced open surgical portosystemic shunt operations as first-line treatment modalities in patients with upper gastrointestinal variceal bleedings related to portal hypertension [1-4,7]. Nevertheless, in case of endoscopic treatment failure (15% of cases in total), a portosystemic shunt operation represents the therapy option of choice [1,2,4,5,8]. In these cases a surgical shunt procedure was indicated for patients with a Child-Pugh A cirrhosis to reduce the incidence of re-bleeding, maintain hepatic function and provide structural stability. In contrast, transjugular intrahepatic portosystemic shunts (TIPS) are indicated for patients suffering from Child B/C cirrhosis and for those scheduled for liver transplantation [1,9,10].

Besides treatment of variceal bleeding episodes, sufficient management of thrombo-embolic complications of the portal venous system is required in patients with PVT suffering from portal hypertension [11]. In about 37% of these patients, PVT is associated with thrombosis of splenic and superior mesenteric veins in combination with a cavernous transformation of the portal vein, and classical portosystemic shunt operations (spleno-renal, porto-caval or mesenterico-caval) are not feasible and/or suitable [12]. In this case, there is need for a different treatment concept.

In the following case report, severe recurrent bleeding episodes from duodenal varices are illustrated in a patient without cirrhosis but suffering from extrahepatic portal hypertension caused by PVT. This case presents a collateral caval shunt operation as an alternative therapy option after failure of endoscopic procedures.

## Case presentation

A 41-year-old male patient was admitted to hospital because of a circulatory collapse with hematemesis. Patient was undergoing phenprocoumon therapy due to deep vein thrombosis 5 years earlier and portal vein thrombosis caused by protein S deficiency 7 years earlier.

The initial basic examination’s result was normocytic anemia and distinct tachycardia (120 beats per minute).

**Figure 1:**
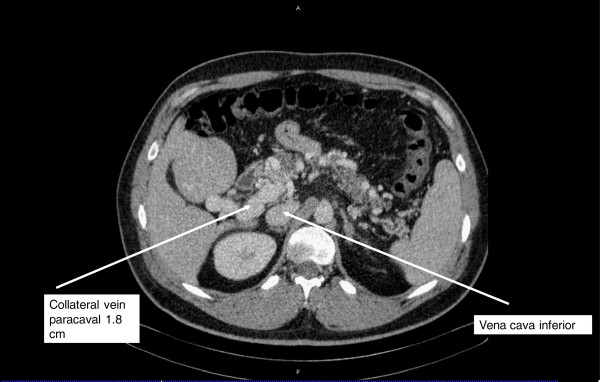
Preoperative computed tomography scan displaying a collateral vein adjacent to the inferior caval vein 1.8 cm in diameter (white arrow) due to portal hypertension caused by extrahepatic portal vein thrombosis.

Blood analysis results showed: Hb 5.1 mmol/l (normal range: 8.1 to 10.7 mmol/l); white blood cell count 3,500/uL (normal range: 4,000 to 9,000/uL); platelet count 11.9 × 10^4^/UL (normal range: 11 to 40 × 10^4^/uL); serum albumin 28.4 g/L (normal range: 35 to 52 g/L); prothrombin time was 31%; and international normalized ratio was 3.91.Emergency esophago-gastroduodenoscopy showed bloody duodenal varices of about 1 cm diameter in the proximal second portion of the duodenum. Bleeding was successfully treated by endoscopic sclerotherapy. Additionally, in endoscopic examination one small esophageal and one isolated gastric varice type I were detected. The patient received blood transfusion with four units of packed red blood cells. Abdominal sonography, as well as computed tomography scan (CT), showed an extrahepatic PVT with involvement/obstruction of the main portal vein including the confluent, proximal part of the right portal branch, suggesting involvement of the proximal part of the upper mesenteric vein. In addition, a cavernous transformation of the portal vein with subsequent extrahepatic portal hypertension was observed. However, there was a collateral vein with approximately 1.8 cm diameter next to the inferior caval vein (Figure 1).

**Figure 2:**
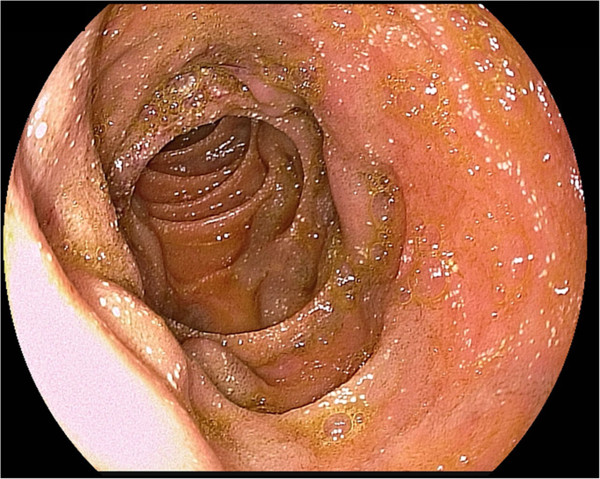
Endoscopic view of nodular varices in the proximal second portion of the duodenum.

Medical therapy was initiated by giving a beta-blocker (propranolol 150 mg per day) to decrease pressure in the portal venous system.Two days after initial endoscopy, a re-endoscopy showed one persisting nodular duodenal varice of about 0.5 to 1 cm diameter with recurrent variceal hemorrhage that responded poorly to sclerotherapy (Figure 2). Another attempt of interventional sclerotherapy in combination with ligation was performed, but was not successful. Due to decreased hemoglobin value, the patient was transfused another two units of packed red blood cells.

On the basis of failed endoscopic attempts to manage bleeding and the long history of PVT with consecutive cavernous transformation (to maybe pose a problem in implementation of TIPS), we scheduled open surgical treatment.

In the context of a distinct PVT with cavernous transformation of the portal venous system and probable inability to reduce pressure in the portal venous system (if classical shunt procedures were to be performed, such as spleno-renal, porto-caval or mesenterico-caval), we decided to apply a different kind of technique.

Consequently, we performed a collateral caval shunt operation with the inferior caval vein and a big collateral vein adjacent to the inferior caval vein.

**Figure 3:**
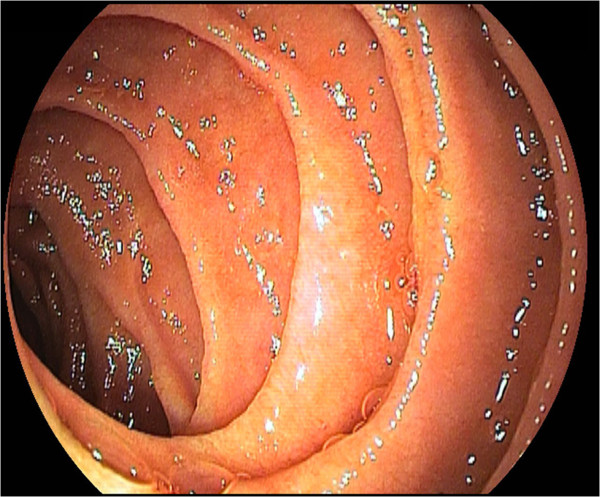
Endoscopic view 12 months after the implementation of the collateral caval shunt with regular relations in the duodenum.

After laparotomy via a mid-transverse Mercedes-type incision in the upper abdomen, a Kocher maneuver was performed, and the caval vein was dissected in a circular shape. The next step repeated this same procedure with the big collateral vein, and then, both vascular structureswere clamped. We continued establishing a side-to-side anastomosis between the collateral vein and inferior caval vein using PDS 4-0 suture.The patient’s postoperative course was uneventful, and no complications were observed. Doppler ultrasound examination showed regular perfusion in the new anastomosis and caval vein. Control endoscopy showed a notable reduction of consistent duodenal varices 2 weeks after surgical treatment. By the end of the follow-up period (12 months), no further bleeding episodes were seen in the patient. Endoscopic examination showed normal mucosal structures in the duodenum (Figure 3). Collaterals along the duodenum and portal vein had disappeared as well, owing to a reduced portal pressure gradient.

## Discussion

As far as we know, this is the first case report describing the treatment with a collateral caval shunt operation of recurrent severe duodenal varice bleeding in a non-cirrhotic patient suffering from portal hypertension and PVT.

This report shows that implementation of a collateral caval shunt might be one good alternative to re-establish decompression in the portal venous system in patients with cavernous transformation of the portal vein and recurrent duodenal variceal bleeding. In this case, we were able to reach a rapid reduction of the pressure gradient in the portal venous system by surgical intervention. By the end of the follow-up period (12 months), a regular structural situation could be observed in the duodenal system. Additionally, peri- and postoperative complications could not be observed and no further bleeding occurred.

Non-cirrhotic, non-tumoral PVT is the second most frequent cause of portal hypertension in the world (5 to 10%). In Asia, extrahepatic PVT is also a common cause for portal hypertension and accountable for up to 30% of total variceal bleeding in comparison to about 10% in the western world [9,12,13]. Etiology of PVT has changed over the last years, accompanied with a variety of causes for non-cirrhotic portal hypertension, such as prothrombotic or thrombophilic disorders, neoplasm, inflammatory lesions (pancreatitis, intra-abdominal sepsis, pylephlebitis of the portal/umbilical veins *etcetera*) on the one hand, and external and operative traumas, polycythemia vera or portal vein obstruction/thrombosis on the other. (Table 1) [1,14].

**Table 1:** **Etiology of portal vein thrombosis modified from Sobhonslidusk A**[14]**and Caronna**[1]

**Thrombophilic disorders**	**Local factors**
**Inherited disorders**	Infections/inflammation
High risk of thrombosis (low prevalence):	Neonatal omphalitis
**Antithrombin** III deficit	Appendicitis
Protein C deficit	Diverticulitis
Protein S deficit	Pancreatitis
	Cholecystitis
Low risk of thrombosis (high prevalence):	Perforated peptic ulcer
Leiden V factor mutation	Tuberculous lymphadenitis
Factor II mutation	
**Acquired disorders**	**Portal vein injury**
Malignancy	Surgical shunts
Myeloproliferative disorders	Splenectomy
Use of oral contraceptives	Abdominal surgery
Antiphospholipid syndrome	Liver transplants
Pregnancy and postpartum	Blunt trauma
Paroxysmal nocturnal	
Hemoglobinuria	
**Mixed disorders**	**Cancer of the abdominal organs Cirrhosis**
Hyperhomocysteinemia	

In most cases, non-cirrhotic patients with chronic PVT do not show any symptoms. They usually first present with complaints due to portal hypertension, such as thrombocytopenia, anemia, splenomegaly or variceal bleeding. In 20 to 55% of all cases, patients with PVT are suffering from gastroesophageal varices [15,16]. Duodenal varices are quite rare and only occur in 0.4% of all patients with portal hypertension [7].

There are different treatment options to manage complications of non- cirrhotic patients suffering from chronic PVT and portal hypertension, such as surgical intervention in contrast to endoscopic and radiological interventions or drug treatment. Besides standard endoscopic interventions, new interventional radiological procedures like TIPS will soon be in the spotlight as an approach for treatment of patients with PVT and/or duodenal varices.

Although TIPS can be performed in patients with severe liver dysfunction, one limitation of this therapy option is that in patients with severe liver atrophy, there is an increased risk for complications such as hepatic encephalopathy or cerebral embolization [7,17,18]. Therefore, in combination with PVT, TIPS has been shown to be successful only in a few cases. Case studies on TIPS are often associated with patients suffering from portal biliopathy and/or portal vein thrombosis or complicated Budd Chiari Syndrome [19,20].

In their large case series of TIPS insertion, Senzalo *et al*. reported on 28 patients suffering from PVT and portal hypertension with a mean follow-up of 18.1 months. Twenty-three of 28 patients showed complete PVT, and 9 of 23 showed cavernous transformation. Eleven of 28 patients suffered from non-cirrhotic portal vein thrombosis. In 19 patients (73%), TIPS insertion was successful. Six patients underwent post-interventional stent revisions, three underwent liver transplantation, and one died from bleeding. Most cirrhotic patients showed a decreased CHILD score [19]. Considering that, some other authors even suggest PVT in combination with cavernous transformation to be a contraindication for TIPS [21,22]. In our case, after conferring with the radiological department, we decided not to perform TIPS for the following reasons: high age of the portal venous thrombus (7 years), already existing total obstruction of the portal vein, including the proximal part of the right branch, involvement of the upper mesenteric vein, and increased risk for the injury of hilar structures by tapping the portal vein. However, TIPS might be an acceptable alternative technique in treatment of selected patients suffering from chronic PVT where the lumen of the thrombosed portal vein is able to be catheterized and the cavernomatous vein is to be dilatated [19].

In view of recent progress in minimally invasive techniques, surgical procedures such as portosystemic shunt operations are about to shrink in importance. Nevertheless, only surgical shunt operations are able to ensure efficient and permanent decompression of the portal venous system in patients suffering from portal hypertension and extrahepatic PVT [9]. Finding the perfect type of shunting procedure to treat patients with extrahepatic PVT in particular has opened the floor for a long-term debate for the last years. Splenorenal shunts (side-to-side or central) and mesocaval shunts have been proved to be an effective tool in management of bleeding episodes [23-25]. In a series, Orloff *et al*. reported 200 patients suffering from portal hypertension caused by PVT, partly treated with splenorenal (134 patients) and partly with mesocaval (66 patients) shunts. Five patients (2.5%), each with a central end-to-side splenorenal shunt, developed shunt thrombosis and had recurrent variceal bleedings. The ten-year-survival rate was 97% versus a 15-year-survival rate of 95%. None of the patients developed any portal-systemic encephalopathy, and all liver function tests (laboratory data, sonography and angiography) were normal [24].

In this context, however, diffuse thrombosis of the entire portal venous system, especially with subsequent development of a cavernous transformation of the portal vein, (portal cavernoma), represents a formidable challenge in patient treatment. Cavernoma usually appear a few days after interruption of portal blood flow, constituting a compensatory mechanism via bypassing the obstructed vein. They are built by portosystemic and peri-/portoportal collaterals [3,12]. Unfortunately, there are only a few case studies reporting some experience with shunt surgery in cases of cavernous transformation of the portal vein [26-28]. In his series, Zhang *et al*. reported surgical treatment in 18 patients with portal cavernous transformation. In 12 of 18 patients, there was history of upper gastrointestinal bleeding, five suffered from splenomegaly and hypersplenism, one suffered from ascites, and six other patients had undergone a ‘splenectomy and disconnection’ in other hospitals. In eight patients a mesocaval shunt was performed, in two patients a splenectomy and disconnection were performed, in three patients a central splenorenal shunt was performed, and in six patients a distal splenorenal shunt operation was performed. There were no deaths or hepatic encephalopathy episodes after operation. After operation portal pressure was reduced significantly, bleeding recurred in two patients (disconnection in one patient and a mesocaval shunt in another one) [27].

However, in this case, we decided not to do a classical shunt procedure like splenorenal or mesenterico-caval for the following reasons: there was well-marked PVT with cavernous transformation of the portal venous system and we objected an involvement of the upper mesenteric vein’s proximal part. In our opinion, the big collateral vein enabled better venous drainage of the portal venous system with consequent achievement of better effects on the portal blood flow than in classical shunt types. Anatomic shape and position of the collateral facilitated anastomizing and made us forego implementing splenorenal shunts.

## Conclusions

Considering the results of this case and other reports, implementation of a collateral caval shunt can be recommended to patients with portal cavernous transformation and recurrent duodenal varices in case classical shunt operations are not suitable or are arguably very difficult ordangerous. Nevertheless, existence of a collateral vein with an acceptable size is an important condition for a successful shunt implementation.

## Consent

Written informed consent was obtained from the patient for publication of this Case report and any accompanying images. A copy of the written consent has been made available for review by the Editor-in-Chief of this journal.

## Abbreviations

CT: computed tomography; PVT: portal vein thrombosis; TIPS: transjugular intrahepatic portosystemic shunts.

## Competing interests

The authors declare that they have no competing interests.

## Authors’ contributions

HMH and PF wrote the paper and designed the study and surgical procedure. MBS, MS, HMH, FK, GW collected data andperformed manuscript and literature research. AH participated in pre- and postoperative episode and follow-up work. SJ gave final approval of the version submitted. All authors contributed to the completion and read and approved the final manuscript.
